# Recent progress of CDK4/6 inhibitors’ current practice in breast cancer

**DOI:** 10.1038/s41417-024-00747-x

**Published:** 2024-02-26

**Authors:** Xueqing Wang, Shanshan Zhao, Qinghan Xin, Yunkun Zhang, Kainan Wang, Man Li

**Affiliations:** 1 Department of Oncology, the Second Hospital of Dalian Medical University, Dalian, China; 2 Department of Breast Surgery, Dalian Municipal Central Hospital, Dalian, China; 3 Department of Pathology, the Second Hospital of Dalian Medical University, Dalian, China

**Keywords:** Targeted therapies, Breast cancer

## Abstract

Dysregulated cellular proliferation represents a hallmark feature across all cancers. Aberrant activation of the cyclin-dependent kinase 4 and 6 (CDK4/6) pathway, independent of mitogenic signaling, engenders uncontrolled breast cancer cell proliferation. Consequently, the advent of CDK4/6 inhibition has constituted a pivotal milestone in the realm of targeted breast cancer therapy. The combination of CDK4/6 inhibitors (CDK4/6i) with endocrine therapy (ET) has emerged as the foremost therapeutic modality for patients afflicted with hormone receptor-positive (HR + )/HER2-negative (HER2-) advanced breast cancer. At present, the Food and Drug Administration (FDA) has sanctioned various CDK4/6i for employment as the primary treatment regimen in HR + /HER2- breast cancer. This therapeutic approach has demonstrated a substantial extension of progression-free survival (PFS), often amounting to several months, when administered alongside endocrine therapy. Within this comprehensive review, we systematically evaluate the utilization strategies of CDK4/6i across various subpopulations of breast cancer and explore potential therapeutic avenues following disease progression during application of CDK4/6i therapy.

## Introduction

Breast cancer ranks as the most common cancer among women globally, accounting for 31% of all female cancers. It stands as the second leading cause of cancer-related fatalities in women [1, 2]. Within the spectrum of breast cancer subtypes, HR+ luminal-like tumors, including luminal A and B, represent the most prevalent category, encompassing 60–70% of all cases. For these tumors, ET serves as the primary first-line treatment in cases of advanced or metastatic breast cancer (MBC). However, the development of ET resistance is an inevitable challenge. Therefore, CDK4/6i have recently emerged as a clinically significant and well-tolerated therapeutic option for patients with MBC.

In estrogen receptor (ER)-positive breast cancer, activation of the ER signaling pathway upregulates the ER-cyclin D-CDK4/6 pathway. Cyclin-CDKs pathway plays a pivotal role in cell-cycle regulation [3]. When bound and activated by cyclin D, CDK4/6 phosphorylates the retinoblastoma protein (Rb), leading to the release of the transcription factor E2F. This promotes the transcription of genes related to the cell cycle, driving the transition of cells from the G1 phase to the S phase [4–8]. Therefore, the inhibition of both CDK4/6 and ER have shown clinical efficacy in ER+ advanced breast cancer [9]. Currently, the USA FDA has approved three CDK4/6i: Palbociclib (Pfizer), Abemaciclib (Eli Lilly), and Ribociclib (Novartis). Furthermore, the China FDA has approved Dalpiciclib (Herngri) for the first- and second-line treatment of HR + /HER2- advanced breast cancer [10]. Considering the effectiveness of CDK4/6i in HR + /HER2- MBC treatment, these drugs are now under investigation in various breast cancer subtypes and different clinical scenarios. For instance, several preclinical studies have indicated that CDK4/6i can boost tumor cell immunogenicity, leading to the exploration of CDK4/6i and Immune checkpoint inhibitor (ICI) combinations [11, 12]. In this review, we compile the most recent preclinical and clinical evidence concerning the application of CDK4/6i in breast cancer and elaborate on the treatment strategies, and advantages of these agents for different patient populations.

## CDK4/6i combined with ET in HR + /HER2- advanced breast cancer

ET was the cornerstone of treatment for HR + /HER2-MBC. In the realm of HR + /HER2- breast cancer treatment, one of the most significant advancements in recent decades is the emergence of CDK4/6i (Table 1), which have proven superior to ET alone when combined with it in the majority of patients, underscoring their pivotal role [8, 13–15].

**Table 1:** Summary of clinical trials evaluating CDK4/6 inhibitors in hormone receptor-positive and HER2-negative metastatic breast cancer.

Trial	Phase	N	Treatments	Primary endpoint Results
PALOMA-2 (NCT01740427)	III	666	Letrozole+Palbociclib vs Placebo	PFS 24.8 vs 14.5 months (HR = 0.58, 95% CI: 0.46–0.72)
MONALEESA-2 (NCT01958021)	III	668	Letrozole+Ribociclib vs Placebo	PFS 25.3 vs 16.0 months (HR = 0.568, 95% CI: 0.457–0.704)
MONARCH-3 (NCT02246621)	III	493	NSAI+Abemaciclib vs Placebo	PFS 28.18 vs 14.76 months (HR = 0.540, 95% CI: 0.418–0.698)
MONALEESA-7 (NCT02278120)	III	672	NSAI/Tamoxifen+Ribociclib+goserelin vs Placebo	PFS 23.8 vs 13.0 months (HR = 0.55, 95% CI: 0.44–0.69)
DAWNA-2 (NCT03966898)	III	456	NSAI+Dalpiciclib vs Placebo	PFS 30.6 vs 18.2 months (HR = 0.51, 95% CI: 0.38–0.69)
MONALEESA-3 (NCT02422615)	III	726	Fulvestrant+Ribociclib vs Placebo	PFS in patients receiving first-line treatment 33.6 vs 19.2 months (HR = 0.55, 95% CI: 0.42–0.72)
FLIPPER (NCT02690480)	II	189	Fulvestrant+Palbociclib vs Placebo	mPFS 31.8 vs 22.0 months (HR = 0.55, 95% CI: 0.34–0.78)
PARSIFAL (NCT02491983)	II	486	Palbociclib+Fulvestrant vs Letrozole	PFS 27.9 vs 32.8 months (HR = 1.13, 95% CI: 0.89–1.45)
MONARCH-2 (NCT02107703)	III	669	Fulvestrant+Abemaciclib vs Placebo	PFS 16.4 vs 9.3 months (HR = 0.553, 95% CI: 0.449–0.681)
MONARCH plus (NCT02763566)	III	207	NSAI+Abemaciclib+ vs Placebo	PFS NR vs 14.7 months (HR = 0.499, 95% CI: 0.346–0.719)
		104	Fulvestrant+Abemaciclib vs Placebo	PFS 11.5 vs 5.6 months (HR = 0.376, 95% CI: 0.240–0.588)
PALOMA-3 (NCT01942135)	III	105	Fulvestrant+Palbociclib vs Placebo	PFS in premenopausal women age ≤50 years 9.5 vs 5.6 months (HR = 0.50, 95% CI: 0.29–0.87)
LEONARDA-1 (NCT05054751)	III	275	Fulvestrant+Lerociclib vs Placebo	PFS 11.07 vs 5.49 months (HR = 0.458, 95% CI: 0.317–0.661)
MAINTAIN (NCT02632045)	II	119	Fulvestrant/Exemestane+Ribociclib vs Placebo	PFS 5.29 vs 2.76 months (HR = 0.57, 95% CI: 0.39–0.85)
BioPER (NCT03184090)	II	33	Palbociclib+ET of physician’s choice	CBR 34.4% (95% CI: 18.6–53.2); 13.0% of tumors (95% CI: 5.2–27.5) showed loss of Rb protein expression
RIGHT (NCT03839823)	II	222	Ribociclib+goserelin+NSAI vs chemotherapy	PFS 24.0 vs 12.3 months (HR = 0.54, 95% CI: 0.36–0.79)

*PFS* progression-free survival, *mPFS* median progression-free survival, *NSAI* Nonsteroidal aromatase inhibitors, *CBR* clinical benefit rate, *ET* endocrine therapy, *HR* hazard ratio, *CI* confidence interval.

### Application in Endocrine-sensitive population

In clinical practice, “endocrine-sensitive population” can be defined as follows: (1) Endocrine-sensitive patients experiencing ET, denoting disease relapse after finishing adjuvant ET more than one year; (2) Endocrine-naive patients, encompassing individuals with recurrent advanced breast cancer who have not previously undergone ET or received adjuvant ET following radical surgery [16, 17]. The PALOMA-2 (77.7% of endocrine-sensitive population), MONALEESA-2 (98% of endocrine-sensitive population), and MONARCH-3 (100% of endocrine-sensitive population) trials assessed the clinical impact of three CDK4/6i—Palbociclib, Ribociclib, and Abemaciclib—in combination with aromatase inhibitor (AI). All of them exhibited significant improvements in PFS and objective response rate (ORR) for postmenopausal women with HR + /HER2- advanced breast cancer, compared to AI monotherapy [2, 18–20]. As for premenopausal women, MONALEESA-7 trial showed that ribociclib plus AI + goserelin also had a superior clinical benefit [21]. The positive PFS and overall survival (OS) results further strengthen AI’s role as a partner in first-line treatment of CDK4/6i [22, 23]. In September 2022, the DAWNA-2 trial released fresh findings at the European Society for Medical Oncology (ESMO), comparing Dalpiciclib plus AI with placebo for endocrine-sensitive HR + /HER2- advanced breast cancer. All patients can benefit from this combination therapy, regardless of their menopausal status. The study reported a median PFS (mPFS) exceeding 30 months, along with a substantial 49% reduction in the risk of disease progression or mortality. Notably, this represents a new PFS and ORR record for the combination of CDK4/6i and AI in the first-line treatment of advanced HR + /HER2- breast cancer, further solidifying its role as the first-line therapy for endocrine-sensitive patients with HR + /HER2- advanced breast cancer [24].

The MONALEESA-3 study (51% of endocrine-sensitive population) recommends Fulvestrant as another partner for CDK4/6i in the treatment of HR + /HER2- advanced breast cancer. Results indicated significant improvements in both PFS (ribociclib vs placebo group: 33.6 vs 19.2 months) and median OS (mOS) (mOS in the ribociclib group was not reached (NR), 95% CI 59.9-NR months; mOS in placebo group: 51.8 months, 95% CI 40.4-57.6 months) [25–28]. Additionally, the GEICAM/2014-12 study (FLIPPER) presented at the 2020 ESMO also focused on evaluating palbociclib in combination with fulvestrant as a first-line treatment for postmenopausal patients with endocrine-sensitive advanced breast cancer. The study demonstrated that this combination significantly improved PFS (palbociclib vs placebo group: 31.8 vs 22.0 months) and ORR (palbociclib vs placebo group: 68.3% vs 42.2%), further supporting its effectiveness in HR + /HER2- advanced breast cancer patients [29]. Additionally, the study’s patient-reported outcomes (PROs) indicated that health-related quality of life (HRQoL) was well-maintained, suggesting a favorable balance between therapeutic benefits and potential toxicities [30].

Recent research has focused on selecting the most effective endocrine partner for CDK4/6i in postmenopausal women with endocrine-sensitive, HR + /HER2- advanced breast cancer. The PARSIFAL trial, as the first head-to-head comparative trial, examined the use of palbociclib with either fulvestrant or letrozole in these patients. While the study demonstrated the favorable safety profiles of both palbociclib combinations, it did not show a significant difference in PFS (fulvestrant-palbociclib vs letrozole-palbociclib group: 27.9 vs 32.8 months; HR = 1.13; 95% CI, 0.89-1.45; *P* = 0.32) or other key outcomes (3-year OS, ORR, and clinical benefit rate, CBR) [31, 32].

In summary, CDK4/6i combinations stand as the primary choice for first-line endocrine therapy in endocrine-sensitive HR + /HER2- advanced breast cancer. The choice between combining CDK4/6i with AI and fulvestrant in the first-line setting remains a subject of ongoing research. Currently, the body of evidence supporting CDK4/6i combined with AI as a first-line treatment surpasses that for fulvestrant. In clinical practice, a personalized and comprehensive treatment strategy should consider factors such as patients’ prior adjuvant endocrine therapy, economic considerations, safety profiles, and the potential need for genetic testing.

### Application in Endocrine-resistant population

Various clinical guidelines provide similar recommendations for the current approach to treating endocrine-resistant breast cancer. Endocrine resistance refers to resistance to current drugs, not necessarily future ones. Hence, even patients with endocrine resistance can rechallenge with endocrine therapy. Among the numerous endocrine treatment options, CDK4/6i are unquestionably essential.

In MONARCH-2, a phase III trial, patients of HR + /HER2- advanced breast cancer (including 24.9% patients with primary endocrine resistance) received fulvestrant plus abemaciclib or a placebo. Abemaciclib increased mPFS from 7.9 to 16.3 months and mOS from 31.5 to 38.7 months. However, the survival benefit wasn’t statistically significant in the primary resistance group due to the small sample [33]. The evidence supporting CDK4/6i for endocrine-resistant population is limited due to the lack of data from a significant portion of the global population. The MONARCH plus trial, primarily conducted in the Chinese population with HR + /HER2- advanced breast cancer, randomized patients to receive anastrozole/letrozole, fulvestrant plus abemaciclib, or a placebo. In cohort B of the MONARCH plus trial, 36 patients (34.6% of the experimental group, higher than in MONARCH-2) had primary resistance to ET. While OS data were not fully developed in the intention-to-treat (ITT) population, there were observable survival benefits in the primary endocrine-resistant group (HR = 0.348, 95% CI: 0.165–0.734) [34]. However, the clinical benefits of CDK4/6i plus ET therapy for endocrine-resistant population in other trials, MONALEESA-3 (ribociclib + fulvestrant) and PALOMA-3 (palbociclib + fulvestrant), are not observed [35–38]. Based on current clinical trial results, abemaciclib is the preferred option for those with primary endocrine resistance, whereas ribociclib or palbociclib does not provide advantages for individuals with primary endocrine resistance. Notably, Dalpiciclib has not been subject to clinical study focusing on endocrine-resistant population. Recently, lerociclib, a novel CDK4/6i, demonstrates superior efficacy in patients with endocrine resistance. The LEONARDA-1, a phase 3 trial, assessed lerociclib plus fulvestrant versus a placebo in patients who had experienced relapse or progression on prior ET. Among these patients, 25.5% exhibited primary endocrine resistance (24.8% in the lerociclib + fulvestrant group versus 26.1% in the placebo + fulvestrant group). The results demonstrated a significantly prolonged PFS in the lerociclib group compared to the placebo group (HR = 0.45, *P* < 0.001). Efficacy remained consistent across all subgroups, including those with primary endocrine resistance, showing a significantly reduced risk of disease progression or death with lerociclib (HR = 0.374, 95% CI: 0.182–0.769) [39].

Rechallenging treatment with an alternative CDK4/6i after disease progression on prior CDK4/6i therapy has been recommended by both domestic and international guidelines. A retrospective analysis of 87 patients with HR+ advanced breast cancer at 6 U.S. cancer centers revealed that using abemaciclib following palbociclib therapy resulted in a mPFS of 5.3 months and an OS of 17.2 months, suggesting potential benefits for heavily pretreated patients [40]. The MAINTAIN trial, a phase II study in advanced HR + /HER2- breast cancer, assessed fulvestrant or exemestane combined with ribociclib or placebo. Notably, 84% of patients had previously received palbociclib, allowing for potential rechallenge with ribociclib-based endocrine therapy. Results presented at the 2022 ASCO Annual Meeting indicated a mPFS of 5.29 months with ribociclib compared to 2.76 months with a placebo. This represents the first prospective randomized controlled study demonstrating the benefit of switching to another CDK4/6i in advanced HR + /HER2- breast cancer patients [41]. However, the approach may not be effective if patients exhibit Rb loss in their tumors, as suggested by the BioPER trial [42]. Currently, there is limited data available on cross-line therapy following CDK4/6i progression, emphasizing the need for large, randomized trials in the future.

### Application in patients with bone metastases

Bone metastases are common in breast cancer, with nearly 70% of advanced breast cancer patients developing bone metastasis (BM) [43, 44]. These metastases often result in skeletal-related events (SREs), such as pain, pathological fractures, and spinal cord or spinal nerve compression, significantly impacting patients’ quality of life and OS [45, 46]. Clinical studies indicate that HR + /HER2- breast cancer has a higher propensity for BM [47]. Recent evidence suggests that CDK4/6i provide a PFS benefit, particularly in breast cancer patients with bone-only disease, leading to extended disease control and improved survival [48, 49].

The PALOMA-2, MONALEESA-2, and MONARCH-3 trials assessed the efficacy of combining ET with CDK4/6i (palbociclib, ribociclib, abemaciclib) as a first-line treatment for HR + /HER2- MBC, including subgroup analyses on patients with exclusive bone metastases [18, 19, 36, 50–52]. In the PALOMA-2 trial, the combination of palbociclib and AI significantly extended PFS to 27.6 months, reducing the risk of disease progression by 44% in the overall population. The most significant benefit was observed in patients with BM (mPFS: 36.2 months, reduction in the risk of disease progression: 59%) [53]. In the meanwhile, both the MONALEESA-2 and MONARCH-3 trials showed a relative PFS benefit in patients with exclusive bone involvement during exploratory subgroup analyses of the combination of abemaciclib and AI (MONALEESA-2: HR = 0.69, 95% CI: 0.38–1.25; MONARCH-3: HR = 0.58, 95% CI: 0.27–1.25). However, these findings did not achieve statistical significance due to the fact that mPFS was not reached in both groups [2, 54]. In a meta-analysis of seven Phase III randomized controlled trials (RCTs, including PALOMA-2, MONALEESA-2, MONALEESA-7, MONARCH-3, FALCON, SWOG, and FACT), the objective was to determine whether CDK4/6i should serve as the first-line treatment for all patients with HR + /HER2- MBC. The findings indicated a significant enhancement in both PFS and ORR when CDK4/6i were administered to patients with BM [55]. Furthermore, all three classic CDK4/6i exhibited comparable efficacy and had the potential to mitigate disease progression in breast cancer patients with BM [56–58].

The PALOMA-3, MONALEESA-3, and MONARCH-2 trials investigated the suitability of CDK4/6i as a treatment option for patients with endocrine-resistant breast cancer and BM. They showed that CDK4/6i plus fulvestrant significantly improved PFS in patients with bone-only metastases (PALOMA-3: HR = 0.43, 95% CI: 0.28–0.67; MONALEESA-3: HR = 0.379, 95% CI: 0.234–0.613; MONARCH-2: HR = 0.543, 95% CI: 0.355–0.833) [27, 58–60]. In conclusion, both endocrine-sensitive and endocrine-resistant HR + /HER2- breast cancer patients with BM can benefit from CDK4/6i treatment.

### Application in patients with visceral metastases

Visceral metastasis (VM), occurring in over 50% of advanced HR + /HER2- breast cancer patients, is an independent poor prognosis risk factor [61–63]. Current clinical practice shows no significant improvement with endocrine monotherapy in VM patients [64–67]. A meta-analysis suggests that VM patients benefit from ET combined with CDK4/6i, while endocrine monotherapy offers limited advantages [68]. Various CDK4/6i combined with AI provide remarkable benefits in endocrine-sensitive patients. Trials like PALOMA-2 (palbociclib plus letrozole, 394 VM patients), MONARCH-3 (abemaciclib plus anastrozole/letrozole, 261 VM patients), MONALEESA-2 (ribociclib plus letrozole, 393 VM patients), and DAWNA-2 (dalpicilib plus letrozole/anastrozole, 277 VM patients) have shown that CDK4/6i combined with AI significantly extend PFS in VM subgroups (HR = 0.561–0.63, 95% CI: 0.408–0.90) [2, 18, 19, 24]. Furthermore, the combination of CDK4/6i and fulvestrant is effective in endocrine-resistant populations. Trials such as PALOMA-2 (311 VM patients), MONARCH-2 (373 VM patients), MONALEESA-3 (439 VM patients), and DAWNA-1 (217 VM patients) have included VM patients. The addition of palbociclib, abemaciclib, ribociclib, or dalpicilib to fulvestrant has significantly improved both PFS (HR = 0.47–0.675, 95% CI: 0.33–0.779) and OS (HR = 0.675, 95% CI: 0.511–0.891) [18, 24, 25, 27, 33, 58]. Thus, endocrine resistance is not a barrier for VM patients, and combining CDK4/6i with fulvestrant presents a promising strategy. Notably, the toxicity of CDK4/6i should be taken into consideration when administering them to patients with VM. Neutropenia, diarrhea, Corrected QT intervals (QTc) prolongation and hepatobiliary toxicity are the key adverse events. As for patients with liver metastasis, the AST/ALT examination is necessary to be performed before CDK4/6i administration.

Additionally, it’s crucial to assess the specific organ sites, organ function, and bone marrow status when considering CDK4/6i combination therapies for VM patients. While the FALCON study’s VM subgroup analysis didn’t show a significant advantage of fulvestrant over anastrozole [64], a retrospective study indicated that patients with non-liver visceral metastases had better therapeutic responses to fulvestrant compared to those with liver metastases across all treatment lines [69]. These findings emphasize the importance of considering specific metastatic sites as independent prognostic factors when making treatment decisions, including rare locations like the brain, meninges, bone marrow, and spinal cord. Such considerations can impact the choice of CDK4/6i. Moreover, it’s essential to avoid CDK4/6i that may exacerbate liver dysfunction in clinical practice to prevent drug-induced liver injury [70, 71]. Evaluating the patient’s baseline bone marrow function is necessary to determine whether they can tolerate the hematologic side effects of CDK4/6i in cases of bone marrow metastases [72, 73]. For patients with brain or meninges metastases, it’s advisable to consider the blood-brain barrier penetration effects of different CDK4/6i based on available exploratory data [74].

Breast cancer with visceral crisis (VC) refers to patients experiencing both VM and the associated symptoms and signs. The latest definition of VC, according to the ESO-ESMO 5th Advanced Breast Cancer (ABC5) guideline, characterizes it as “severe organ dysfunction assessed through signs, symptoms, laboratory tests, and rapid disease progression” [75]. Historically, chemotherapy (CT) was recommended for this urgent, life-threatening condition. However, some patients couldn’t endure the strong cytotoxic effects of CT due to poor liver function [76–78]. The introduction of CDK4/6i has overturned the notion of slow-acting ET drugs and has brought forth an optimal therapeutic strategy with good efficacy and fewer adverse events [79]. Recent findings indicate that CT does not offer superior outcomes compared to CDK4/6i when used in combination with ET for first/second-line treatment of HR + /HER2- MBC, especially concerning PFS [80]. Real-world data also supports the use of CDK4/6i in cases of VC, showing a significant 5-month improvement in OS compared to CT [81]. Moreover, a recent case report has questioned the traditional approach of using CT for all patients with VC, proposing the possibility of employing CDK4/6i in poor prognostic scenarios [82]. The RIGHT Choice, a randomized, phase II, open-label, multicenter clinical trial presented at the 2022 San Antonio Breast Cancer Symposium (SABCS), offered the first head-to-head comparison of CDK4/6i plus ET versus CT in advanced HR + /HER2- breast cancer with life-threatening VC. With a median follow-up of 24.1 months, ribociclib plus ET led to a significant improvement in mPFS, extending it from 12.3 months to 24.0 months compared to the CT group. This approach also notably reduced the risk of progression or death (HR = 0.54; 95% CI: 0.36–0.79; *P* = 0.0007). Comparable improvements were observed in ORR (65.2% vs. 60.0%), CBR (80.4% vs. 72.7%), and time to remission (TTR) (4.9 months vs. 3.2 months; HR = 0.78, 95% CI: 0.56–1.09) in the ribociclib group compared to CT. Hence, ribociclib combined with ET emerges as a preferred choice for patients with VC [83]. These findings collectively emphasize the potential of CDK4/6i in the management of advanced breast cancer, offering more tailored and effective treatment options for patients in VC.

## CDK4/6i in combination with HER2-targeted therapy

Compared to HR + /HER2- breast cancer, HR + /HER2+ breast cancer exhibits a more aggressive nature and diminished sensitivity to ET and HER2-targeted therapy, resulting in a poorer prognosis. Recent studies have unveiled the underlying mechanisms. The HER2 pathway has been linked to heightened endoplasmic reticulum phosphorylation and has demonstrated endoplasmic reticulum’s role in fostering resistance to ET [84, 85]. Moreover, CDK4/6, positioned downstream of the HER2 signaling pathway, plays a pivotal role in conferring resistance to HER2-targeted therapy [86]. Hence, CDK4/6 emerges as an attractive target to enhance the survival prospects of HR + /HER2+ breast cancer patients. Research has shown that HER2 significantly boosts CDK4/6 activity through phosphorylated Rb (pRb) in HER2+ breast cancer, suggesting the potential responsiveness of HER2+ breast cancer to CDK4/6i [87]. The combination of palbociclib with either tamoxifen or trastuzumab has demonstrated inhibitory effects in both ER+ and HER2+ cell lines [88]. Preclinical data support the notion that CDK4/6i can overcome resistance to HER2-targeted therapies, thereby delaying recurrence in HER2+ breast cancer patients [89]. Moreover, several clinical trials have substantiated the antitumor efficacy of CDK4/6i in HR + /HER2+ breast cancer (Table 2). The monarcHER and PATRIICA trials have revealed a synergistic benefit in the co-inhibition of CDK4/6 and HER2 for the treatment of advanced HR + /HER2+ breast cancer [90, 91]. The DAP-Her-01 study was the first to assess the efficacy and safety of orally administered dalpiciclib and HER2 tyrosine kinase inhibitor (TKI) pyrotinib as a first-line treatment for HER2+ advanced breast cancer. After a median follow-up of 25.9 months, the ORR was 70% (95% CI: 53.5–83.4%), with a mPFS of 11.0 months (95% CI: 7.3–19.3 months), while OS data were still pending. Additionally, findings from the DAP-HER-01 study have indicated that the combination of dalpiciclib and pyrotinib holds promise as an optimal strategy for HER2+ breast cancer patients with brain metastases [92]. Notably, nearly half of patients with HER2 + MBC will eventually develop brain metastases [93]. Consequently, identifying novel systemic therapies capable of effectively penetrating the blood-brain barrier (BBB) and concurrently targeting extracranial disease is an appealing strategy. Previous studies have suggested that abemaciclib attains higher central nervous system concentrations at a potentially lower dose compared to palbociclib [94]. Currently, a trial (DAP-HER-02, NCT05328440) investigating first-line treatment for HER2+ advanced breast cancer with a combination of dalpiciclib and pyrotinib, along with fulvestrant or inetetamab, is currently underway.

**Table 2:** Summary of clinical trials evaluating CDK4/6 inhibitors in combination with HER2-targeted therapy or immunotherapy.

Trial	Phase	N	Treatments	Primary endpoint Results
monarcHER (NCT02675231)	II	325	Abemaciclib+Trastuzumab+Fulvestrant (A) Abemaciclib+Trastuzumab(B) Chemotherapy+Trastuzumab(C)	PFS between A and C 8.3 vs 5.7 months (HR = 0.673, 95% CI: 0.45-1.00)
				PFS between B and C 5.7 vs 5.7 months (HR = 0.94, 95% CI: 0.64-1.38)
PATRIICA (NCT02448420)	II	71	Palbociclib+Trastuzumab (ER-) / (ER + ) Palbociclib+Trastuzumab+letrozole(ER + )	PFS6, n (%, 95% CI) 5 (33.3, 10.8–77.8) / 12 (42.9, 24.5–62.86) 13 (46.4, 27.5–66.1)
DAP-HER-01 (NCT04293276)	II	41	Dalpiciclib+Pyrotinib	mPFS in evaluable patients: 11.0 months (95% CI: 7.3-19.3)
DAP-HER-02 (NCT05328440)	II		Dalpiciclib+Pyrotinib+Fulvestrant vs Inetetamab	Ongoing
NCT02779751	Ib	54	Abemaciclib+Pembrolizumab+Anastrozole vs Abemaciclib+Pembrolizumab	Neutropenia (30.8/28.6%), AST increase (34.6/17.9%), ALT increase (42.3/10.7%), and diarrhea (3.8/10.7%) were the most frequent grade ≥3 adverse events
CheckMate 7A8 (NCT04075604)	Ib/II	21	Palbociclib 125-mg/ 100-mg	DLTs 2 (22.2%)/ 0 patient
	Ib	6	Ribociclib 400 mg/ 600 mg + spartalizumab 400 mg	no DLTs were observed
		4	Ribociclib 600 mg+spartalizumab 400 mg	1/3 experienced a DLT of grade 3 atrial fibrillation and flutter
NEWFLAME	II	17	Nivolumab+Abemaciclib+Fulvestrant vs Letrozole	ORR 54.5% (6/11) vs 40.0% (2/5)

*PFS* progression-free survival, *PFS6* progression-free survival rate at 6 months, *mPFS* median progression-free survival, *DLTs* dose-limiting toxicities, *ORR* objective response rate, *HR* hazard ratio, *CI* confidence interval.

## CDK4/6i in combination with immunotherapy

CDK4/6i have demonstrated an enhancement of the antitumor immune response. Beyond their direct impact on tumor cells, they can influence immune cells within the tumor microenvironment. This influence plays a pivotal role in the proliferation and differentiation of T-cells, as well as in the chemokine-mediated recruitment of T-cells to mammary tumors. Preclinical studies have confirmed that CDK4/6i selectively inhibits regulatory T cells (Tregs) and dendritic cells (DC), which can induce immunological tolerance and suppress the immune response [95, 96]. These findings suggest that the use of CDK4/6i in treating tumors may render them more responsive to immunotherapy.

Currently, there are ongoing preclinical and clinical trials investigating the combination of CDK4/6i with immune checkpoint blockade (ICB) therapy for breast cancer. However, initial results indicate that adding ICB does not significantly enhance responses compared to CDK4/6i monotherapy (Table 2). For instance, the combination of abemaciclib with the anti-programmed death-1 (PD-1) antibody pembrolizumab, with or without ET, in patients with HR + /HER2- MBC who have not been previously exposed to CDK4/6i did not demonstrate a clear improvement when compared to abemaciclib monotherapy [97]. Similarly, combining palbociclib or ribociclib with the PD-1 inhibitor did not yield significant efficacy [98, 99]. Recently, the NEWFLAME trial, a phase II study, assessed the effectiveness of combining nivolumab, abemaciclib, and endocrine therapy (fulvestrant or letrozole) in patients with HR + /HER2- MBC. While the results indicated an antitumor response, they were accompanied by a high incidence of severe immune-related adverse events, which does not warrant further investigation [100].

## Mechanism of action of CDK4/6 inhibitors

Mechanistically, ATP-competitive CDK4/6 dual inhibitors such as abemaciclib, palbociclib and ribociclib, block CDK4/6, stop the phosphorylation of Rb and arrest G1 cell cycle development of tumor cells. In terms of molecular structure, the substituents in hydrophobic post-pocket of abemaciclib (2 fluorine atoms) is smaller than that of palbociclib and ribociclib (dimethylatmide and acethal groups) [101]. This difference may result in lower kinase selectivity of abemaciclib compared to the other two CDK4/6i. It also leads to different adverse reactions of the three CDK4/6i. Abemaciclib stands out among CDK4/6 inhibitors with its higher CDK4/6 inhibitory constant (Ki) ATP ratio, emphasizing its stronger selectivity for CDK4 but lower selectivity for CDK6. This unique attribute not only enhances its effectiveness in inhibiting breast cancer cell proliferation but also reduces the risk of bone marrow toxicity. Interestingly, the most common adverse event in palbociclib and ribociclib is neutropenia [102]. Additionally, palbociclib and ribociclib can only inhibit CDK4 and CDK6, whereas abemaciclib also has an activity against CDK9, which may related to intestinal toxicity induced by abemaciclib specifically. Transaminase elevation and QT interval prolongation are also notable toxicities reported with ribociclib [103]. Due to extensive hepatic metabolism through the CYP3A4A pathway, CDK4/6i have been shown to have a serious effect on liver enzymes. Ribociclib is notorious for causing elevated transaminases with grade 3-4 toxicity occurred 7%-10% of the time [104]. Furthermore, the mechanism of QT prolongation is reported that ribociclib inhibits one or more kinases, which may modify ion channel protein function and potassium [105]. Overall, research on CDK4/6i toxicity is essential to strike a balance between the benefits of treatment and the potential risks to a patient’s health.

## Summary

**Fig. 1:**
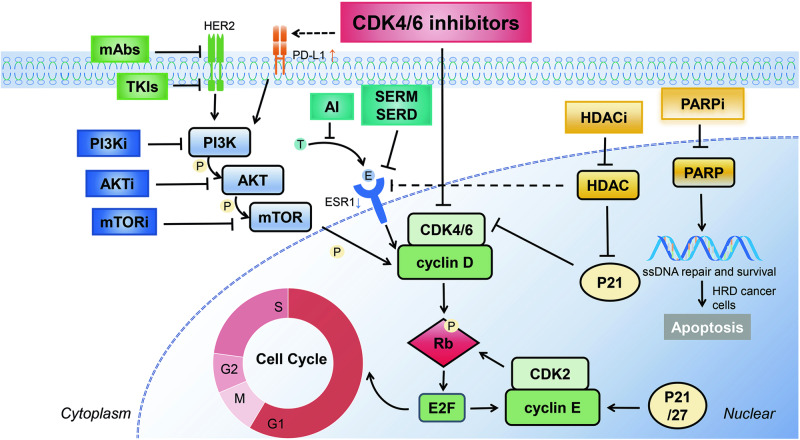
Regulation of cell cycle in breast cancer in CDK4/6 inhibitors. AI, aromatase inhibitor (anastrozole, letrozole, exemestane); SERM, selective estrogen receptor modulator (Tamoxifen (TAM)); SERD, selective estrogen receptor down-regulator (fulvestrant); ER, estrogen receptor; E, estradiol; T testosterone; TKIs, tyrosine kinase inhibitors (pyrotinib, lapatinib); mAbs, (trastuzumab, pertuzumab); PI3Ki, Phosphoinositide 3-kinases inhibitor (alpelisib); AKTi, protein-kinase B inhibitor (capivasertib); mTORi, mammalian target of rapamycin inhibitor (everolimus); E2F, a transcription factor; Rb, retinoblastoma protein; HER2, human epidermal growth-factor receptor 2; HDACi, HDAC inhibitor (tucidinostat); PARPi, PARP inhibitor (olaparib, talazoparib); HRD, homologous recombination deficiency; CDK4/6 inhibitors, cyclin-dependent kinase 4/6 (Palbociclib, Ribociclib, Abemaciclib, Dalpiciclib).

The introduction of CDK4/6i has significantly advanced the therapeutic landscape for HR+ breast cancer (Fig. 1). Currently, CDK4/6i combined with ET represent the standard first-line treatment for both premenopausal and postmenopausal women. In the endocrine-sensitive population, combining CDK4/6i with AI or fulvestrant has demonstrated comparable enhancements in PFS and OS. However, in the endocrine-resistant population, the combination of fulvestrant and CDK4/6i has yielded superior clinical benefits.

BM are common in breast cancer, and CDK4/6i show promise in patients with bone-only metastases. However, in cases of VC, which are associated with a less favorable prognosis, endocrine monotherapy offers limited advantages. The decision to use CDK4/6i should be guided by the specific metastatic sites and organ functionality.

Furthermore, preclinical and clinical investigations have indicated that CDK4/6i can augment the response to HER2-targeted therapy. Additionally, CDK4/6i have shown the ability to enhance anti-tumor immune responses. However, ongoing research is evaluating the combination of CDK4/6i with ICB, with initial findings not demonstrating significant benefits. CDK4/6i and ICB monotherapy are shown to have overlapping toxicities, particularly hepatic events and interstitial lung disease/pneumonitis, and adverse events were found to be a leading cause of treatment discontinuation. Recent applications of CDK4/6i in neoadjuvant therapy for breast cancer are the subject of ongoing clinical trials. To summarize, CDK4/6i have revolutionized the management of HR+ breast cancer. These findings emphasize the significance of personalized treatment strategies for breast cancer patients.

## Data Availability

All data generated or analyzed during this study are included in this published article and referenced articles are listed in the References section.
